# Genetic associations of Nrf2-encoding *NFE2L2* variants with Parkinson’s disease – a multicenter study

**DOI:** 10.1186/s12881-014-0131-4

**Published:** 2014-12-12

**Authors:** Malin von Otter, Petra Bergström, Aldo Quattrone, Elvira Valeria De Marco, Grazia Annesi, Peter Söderkvist, Stephanie Bezzina Wettinger, Marek Drozdzik, Monika Bialecka, Hans Nissbrandt, Christine Klein, Michael Nilsson, Ola Hammarsten, Staffan Nilsson, Henrik Zetterberg

**Affiliations:** Institute of Neuroscience and Physiology, Department of Psychiatry and Neurochemistry, The Sahlgrenska Academy at the University of Gothenburg, Blå stråket 15, 413 45 Gothenburg, Sweden; Institute of Neurology, University Magna Graecia, Catanzaro, Italy; Neuroimaging Research Unit, Institute of Molecular Bioimaging and Physiology, National Research Council, Catanzaro, Italy; Institute of Neurological Sciences, National Research Council, Cosenza, Italy; Institute of Molecular Bioimaging and Physiology, Section of Germaneto, National Research Council, Catanzaro, Italy; Division of Cell Biology, Department of Clinical and Experimental Medicine, Linköping University, SE-581 85 Linköping, Sweden; Department of Applied Biomedical Science, Faculty of Health Sciences, University of Malta, Msida, Malta; Department of Pharmacology, Pomeranian Medical University, Powstancow Wlkp. 72, Szczecin, 70-111 Poland; Institute of Neuroscience and Physiology, Department of Pharmacology, the Sahlgrenska Academy at the University of Gothenburg, Box 431, 405 30 Gothenburg, Sweden; Institute of Neurogenetics, University of Luebeck, Luebeck, Germany; Institute of Neuroscience and Physiology, Center for Brain Repair and Rehabilitation, the Sahlgrenska Academy at the University of Gothenburg, Per Dubbsgatan 14, 413 45 Gothenburg, Sweden; Hunter Medical Research Institute, University of Newcastle, Newcastle, Australia; Institute of Biomedicine, Department of Clinical Chemistry and Transfusion Medicine, The Sahlgrenska Academy at the University of Gothenburg, Bruna Stråket 16, 413 45 Gothenburg, Sweden; Institute of Mathematical Sciences, Department of Mathematical Statistics, Chalmers University of Technology, Chalmers tvärgata 3, 412 96 Gothenburg, Sweden; UCL Institute of Neurology, Queen Square, London, WC1N 3BG UK

**Keywords:** Parkinson’s disease, PD, Nrf2, *NFE2L2*, Meta-analysis, Multicenter, SNP, Haplotype, Risk factor

## Abstract

**Background:**

The transcription factor Nrf2, encoded by the *NFE2L2* gene, is an important regulator of the cellular protection against oxidative stress. Parkinson’s disease is a neurodegenerative disease highly associated with oxidative stress. In a previously published study, we reported associations of *NFE2L2* haplotypes with risk and age at onset of idiopathic Parkinson’s disease in a Swedish discovery material and a Polish replication material. Here, we have extended the replication study and performed meta-analyses including the Polish material and four new independent European patient-control materials. Furthermore, all SNPs included in the haplotype windows were investigated individually for associations with Parkinson’s disease in meta-analyses including all six materials.

**Methods:**

Totally 1038 patients and 1600 control subjects were studied. Based on previous *NFE2L2* haplotype associations with Parkinson’s disease, five *NFE2L2* tag SNPs were genotyped by allelic discrimination and three functional *NFE2L2* promoter SNPs were genotyped by sequencing. The impact of individual SNPs and haplotypes on risk and age at onset of Parkinson’s disease were investigated in each material individually and in meta-analyses of the obtained results.

**Results:**

Meta-analyses of *NFE2L2* haplotypes showed association of haplotype G**AGC**AAAA, including the fully functional promoter haplotype AGC, with decreased risk (OR = 0.8 per allele, p = 0.012) and delayed onset (+1.1 years per allele, p = 0.048) of Parkinson’s disease. These results support the previously observed protective effect of this haplotype in the first study. Further, meta-analyses of the SNPs included in the haplotypes revealed four *NFE2L2* SNPs associated with age at onset of Parkinson’s disease (rs7557529 G > A, −1.0 years per allele, p = 0.042; rs35652124 A > G, −1.1 years per allele, p = 0.045; rs2886161 A > G, −1.2 years per allele, p = 0.021; rs1806649 G > A, +1.2 years per allele, p = 0.029). One of these (rs35652124) is a functional SNP located in the *NFE2L2* promoter. No individual SNP was associated with risk of Parkinson’s disease.

**Conclusion:**

Our results support the hypothesis that variation in the *NFE2L2* gene, encoding a central protein in the cellular protection against oxidative stress, may contribute to the pathogenesis of Parkinson’s disease. Functional studies are now needed to explore these results further.

**Electronic supplementary material:**

The online version of this article (doi:10.1186/s12881-014-0131-4) contains supplementary material, which is available to authorized users.

## Background

Parkinson’s disease (PD) is a neurodegenerative disease affecting the central nervous system, resulting in motor symptoms such as rigidity, slowness of movement, postural instability and a characteristic resting tremor. The motor symptoms derive from a decrease in the neurotransmitter dopamine (DA), due to death of dopaminergic (DAergic) neurons in the substantia nigra (SN). Even though several PD-causing genes have been identified, the majority of PD patients have an idiopathic form without known cause and the disease process may involve a combination of genetic and environmental factors (reviewed in [1]). PD pathogenesis is known to involve oxidative stress and the SN seems to be especially vulnerable. Incomplete intracellular oxidation of DA in the SN, favored by the presence of ferrous iron, may result in the formation of reactive DA(semi)-quinones, which together with decreased levels of reduced glutathione and chronic inflammation adds to the oxidative stress observed in the PD brain (reviewed in [2,3]).

Nuclear factor erythroid 2 (NF-E2) related factor 2 (Nrf2), a transcription factor encoded by the NF-E2-like 2 (*NFE2L2*) gene, has a key role in the cellular protection against oxidative and electrophilic insults [4]. In the normal state, Nrf2 is kept largely inactive by its repressor protein kelch-like ECH-associated protein 1 (Keap1) [5], which targets Nrf2 for ubiquitin ligation and subsequent degradation by the proteasome [6,7]. When Nrf2 is activated by oxidative or electrophilic stress, it induces transcription of a battery of cytoprotective genes by binding to a specific region in their promoters – the antioxidant response element (ARE) [8,9]. Nrf2 can also be activated by dietary factors, such as sulforaphane or curcumin, and Nrf2-activating substances have been used in a number of pre-clinical PD models to study the effect of Nrf2 upregulation on PD progression with a pharmacological perspective (reviewed in [10,11]. Interestingly, one study has shown that an increased oxidative stress observed in olfactory neurosphere-derived cells from PD patients could be restored by activation of Nrf2 with sulforaphane [12] and curcumin has been shown to protect DAergic SH-SY5Y neurons from 6-Hydroxydopamine toxicity [13]. Another study has shown that upregulation of Nrf2 using potent synthetic Nrf2 activators protects DAergic neurons from degeneration in a 1-methyl-4-phenyl-1,2,3,6-tetrahydropyridine (MPTP) mouse model of PD [14].

Nrf2 is essential in regulation of the cellular redox homeostasis, as it controls rate-limiting steps in the neo-synthesis of glutathione [15], as well as the induction of the antioxidant and neuroprotective enzyme heme oxygenase 1 (HO-1) [16,17]. Another protein regulated by Nrf2 is the highly inducible NAD(P)H dehydrogenase, quinone 1 (NQO1) [18]. Besides its broad general antioxidant activity, NQO1 prevents toxic redox cycling of DA-quinones through a two-electron reduction into stable hydroquinones [19]. NQO1 has been found to be expressed in the SN of PD patients but not in age-matched control subjects [20], suggesting an increased Nrf2 activity in the PD brain. Nrf2 nuclear translocation is enhanced in the PD affected SN [21] and a recent study showing an increased Nrf2 activity in neurons derived from induced pluripotent stem cells (iPSCs) from PD patients [22] indicates a role for Nrf2 activation in the PD pathogenesis. Considering all this, it is feasible that a decreased radical protection due to genetic variation in the *NFE2L2* gene could affect the pathogenesis of PD.

We previously reported that a haplotype in the *NFE2L2* gene, GAAAA consisting of five tag single nucleotide polymorphisms (SNPs), was associated with delayed age at onset (AAO) in a Swedish discovery material and with decreased risk of PD in an independent Polish replication material [23]. In the same study, investigation of three functional SNPs in the human *NFE2L2* promoter, previously shown to influence Nrf2 protein expression [24,25], revealed that the protective haplotype GAAAA was in linkage disequilibrium (LD) with the promoter haplotype AGC, which is part of a fully functional promoter [23].

Here, based on our previous *NFE2L2* haplotype associations with PD in the Swedish Gothenburg (PD-Goth) discovery material, seven *NFE2L2* haplotypes and one individual SNP were investigated for associations with risk and/or AAO of idiopathic PD in meta-analyses including five independent patient-control replication materials. Furthermore, with an exploratory approach despite lack of association with risk in our previous study, all genotyped SNPs were also independently investigated in meta-analyses including the PD-Goth discovery material.

## Methods

### Patient-control materials

In total, 2638 individuals (1038 PD patients and 1600 controls) were included in this study. Subjects originated from six independent research centers in Europe: Italy, Malta, Poland and Germany, as well as two independent patient-control sets from Sweden: Gothenburg (PD-Goth) and Linköping (PD-Link).

#### PD-Goth

PD-Goth was the discovery material in our previous study [23] and included 165 PD patients and 190 control individuals. All participants were of Caucasian origin and patients and control subjects were of similar age. The patients were diagnosed according to the Parkinson’s Disease Society Brain Bank criteria for idiopathic PD, except that presence of more than one relative with PD was not considered a criterion for exclusion. PD patients with an AAO of < 50 years were screened to exclude mutations in the recognized PD-causing genes DJ-1, Parkin, PINK1 and LRRK2 [26,27].

#### Italy

The Italian material included 329 PD patients and 450 control subjects. All participants were of Caucasian origin and the patients were diagnosed according to the Parkinson’s Disease Society Brain Bank criteria for idiopathic PD. The controls were unrelated subjects originating from the same geographical area as the patients and all underwent neurological examination for exclusion of neurological diseases.

#### PD-Link

The PD-Link material included 195 PD patients and 379 control subjects collected as part of the Geoparkinson Study [28]. Blood samples were collected from L-dopa positive PD patients in southeast Sweden visiting the Clinic from Geriatrics and Neurology, University Hospital in Linköping, Sweden. The patients were classified as having Parkinson’s disease or parkinsonism using the United Kingdom Parkinson’s Disease Society Brain Bank clinical diagnostic criteria and a neurologist confirmed the diagnosis at recruitment. Individuals with vascular or drug-induced parkinsonism were excluded from the study, as were those with dementia. In this study, only samples from patients with confirmed PD were used. The controls were unrelated Swedish subjects randomly collected from the normal population in southeast Sweden, the same study base as the patients. The patient and control groups were frequency-matched by age and sex.

#### Malta

The Malta material included 101 PD patients and 313 control subjects collected as part of the Geoparkinson Study [28]. All participants were of Caucasian (Maltese) origin. The patients were classified as having Parkinson’s disease or parkinsonism using the United Kingdom Parkinson’s Disease Society Brain Bank clinical diagnostic criteria. In this study, only samples from patients with confirmed PD were used. The controls were from the community or from out-patients at St Luke’s Hospital, G’Mangia, Malta. They were group-matched to the patients by age and sex.

#### Poland

The Polish material was the replication material in our previous study. It included 192 PD patients and 192 control subjects. All participants were of Caucasian origin from the same geographic area, and were matched by sex. Age at sampling (AAS) was significantly higher in the control subjects than in the PD patients (to minimize the risk of PD development in the controls later in life). The patients fulfilled the Parkinson’s Disease Society Brain Bank criteria for idiopathic PD except for the presence of more than one relative with PD, i.e. all patients with a family history of PD were excluded from the study.

#### Germany

The German material included 56 PD patients and 76 control subjects. All participants were of Caucasian origin and were matched by age and sex. PD was diagnosed by movement disorders specialists according to the Parkinson’s Disease Society Brain Bank criteria for idiopathic PD. The controls were also personally examined and free of any symptoms or signs suggestive of PD.

All materials contained information regarding sex and AAS of patients and control subjects, and AAO of patients (i.e. age at diagnosis (AAD) for Germany, Malta and PD-Link; and age at first symptom (AAFS) for PD-Goth, Poland and Italy). Some materials also included information on family history (FH; one or more 1st degree family member(s) with PD) and smoking status. Demographics are summarized in Table 1.

**Table 1:** **Demographic characteristics of PD patients and control subjects**

**Parameter**	**Sweden PD-Goth** ^**1**^			**Italy**			**Sweden PD-Link**		
	**PD**	**Control**	**p-value**	**PD**	**Control**	**p-value**	**PD**	**Control**	**p-value**
N	165	190	---	329	450	---	195	379	---
Sex (Male)	94 (57.0)	70 (36.8)	<0.001	205 (62.3)	179 (39.8)	<0.001	121 (62.1)	187 (49.3)	0.004^3^
AAS (years)	68.2 ± 8.8	69.1 ± 9.3	0.698	66.5 ± 9.2	57.0 ± 17.4	<0.001	71.4 ± 8.7	67.5 ± 9.7	<0.001^3^
AAO^2^ (years)	59.0 ± 10.2	---	---	60.1 ± 10.1	---	---	63.6 ± 10.0	---	---
	N = 164			N = 328					
FH	15 (9.3)	9 (4.8)	0.096	24 (7.3)	ni	ni	14 (7.2)	15 (4.0)	0.095
	N = 162	N = 189							
Current smoker	9 (8.7)	13 (8.3)	0.897	17 (8.2)	ni	ni	9 (4.6)	56 (14.8)	<0.001
				N = 208			N = 194		
Ever smoked	38 (36.9)	81 (51.6)	0.020	77 (37.0)	ni	ni	63 (32.5)	183 (48.3)	<0.001
				N = 208			N = 194		
**Parameter**	**Malta**			**Poland**			**Germany**		
	**PD**	**Control**	**p-value**	**PD**	**Control**	**p-value**	**PD**	**Control**	**p-value**
N	101	313	---	192	192	---	56	76	---
Sex (Male)	63 (62.4)	197 (62.9)	0.919^3^	117 (60.9)	117 (60.9)	1.000^3^	33 (58.9)	34 (44.7)	0.107^3^
AAS (years)	71.3 ± 10.0	72.7 ± 10.0	0.176^3^	63.7 ± 10.9	72.9 ± 9.9	<0.001^4^	66.0 ± 11.9	57.8 ± 10.8	<0.001^3^
AAO^1^ (years)	64.1 ± 11.6	---	---	55.2 ± 10.9	---	---	58.7 ± 12.5	---	---
	N = 76						N = 54		
FH	13 (12.9)	8 (2.6)	<0.001	ni	ni	ni	ni	ni	ni
Current smoker	3 (3.0)	25 (8.1)	0.076	ni	ni	ni	ni	ni	ni
		N = 308							
Ever smoked	31 (30.7)	149 (47.6)	0.003	ni	ni	ni	ni	ni	ni

Data are presented as absolute numbers (%) or mean ± SD. p-values were calculated with Pearson χ^2^-test for categorical parameters and Mann–Whitney U test for continuous parameters. AAS: age at sampling; AAO: age at onset; AAFS: age at first symptom; AAD: age at diagnosis; ni: no information available. FH: family history (one or more 1st degree family member(s) with PD). N-numbers are shown if data was not available for all subjects in the analysis.

^1^Discovery material on which the replication analyses in this study are based.

^2^AAO is: AAFS for Italy, PD-Goth and Poland; AAD for Germany, Malta and PD-Link.

^3^The results are expected to be similar between the groups due to the applied matching in the original study design. Significant p-values are likely a result of not including all samples from the original studies.

^4^This result is expected due to the original study design.

### SNP selection

**Figure 1:**
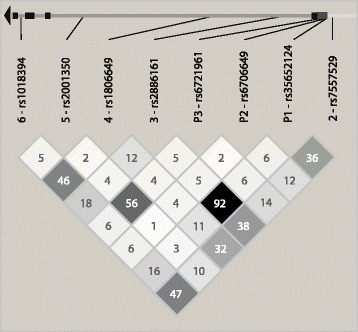
**Schematic** ***NFE2L2*** **gene positions of the studied SNPs and their LD-patterns, showing r** ^**2**^ **-values as 100r** ^**2**^ **.**

Selection of common SNP genotyping data covering *NFE2L2* for the European material CEU (Utah residents with ancestry from Northern and Western Europe) were performed when designing our previous study [23]. In brief, SNP genotyping data was downloaded from the International Haplotype Mapping Project web site (www.hapmap.org) [29] and processed using the Haploview software [30]. LD blocks were constructed according to Gabriel *et al.* [31] and tag SNPs assigned using the tagger function [30]. A minor allele frequency of ≥ 5% and pair-wise tagging with a minimum r^2^ of 0.80 were applied to capture the common SNPs within the block covering the *NFE2L2* gene. In the same study promoter SNPs were chosen due to their reported functional effect on Nrf2 transcription [24,25]. The analyzed SNPs, included in the haplotype window previously associated with PD [23], were: five tag SNPs (rs7557529, rs2886161, rs1806649, rs2001350 and rs10183914, here SNPs 2 – 6) and three functional promoter SNPs [24,25] (rs35652124, rs6706649 and rs6721961, here SNPs P1–P3 [23]), (Figure 1, Table 2).

**Table 2:** **Overview of the studied SNPs**

**SNP**	**rs-ID**	**Genome position**	**Alleles d > D**	**Gene location**	**SNP type**	**Taqman assay**
***NFE2L2***		**Chr:2(−)**				
2	rs7557529	177843343	G > A	5′-region	–	C__436313_10
P1	rs35652124	177838319	A > G	Promoter (−653)	Regulatory^1^	Sequencing^2^
P2	rs6706649	177838317	G > A	Promoter (−651)	Regulatory^1^	Sequencing^2^
P3	rs6721961	177838283	C > A	Promoter (−617)	Regulatory^1^	Sequencing^2^
3	rs2886161	177836085	A > G	Intron 1	–	C__351881_10
4	rs1806649	177826398	G > A	Intron 1	–	C_11634983_10
5	rs2001350	177808671	A > G	Intron 1	–	C_11634985_10
6	rs10183914	177805912	G > A	Intron 3	–	C__157561_10

The SNPs analyzed in the study are numbered according to gene location in reference [23]; SNPs 2 – 6 are tag SNPs; SNPs P1–P3 are functional promoter SNPs. Genome positions were obtained from the CEU population of the HapMap Genome Browser (Phase 1 & 2 full dataset). Alleles are given according to the sense sequence of the gene.

^1^See reference [25].

^2^See reference [23].

### Tag SNP genotyping and promoter sequencing

A detailed description of tag SNP genotyping and sequencing of promoter SNPs is provided in our previous study [23]. In brief, all SNPs were genotyped using genomic DNA extracted from blood. All tag SNPs were genotyped using TaqMan Allelic Discrimination [32] with TaqMan® Pre-Designed SNP genotyping assays or TaqMan® Custom Made SNP genotyping assays (Applied Biosystems, Foster City, CA, USA). The promoter SNPs were genotyped by sequencing, amplifying a 423 bp region of the *NFE2L2* promoter (forward primer 5’-GACCACTCTCCGACCTAAAGG-3’, reverse primer 5’-CGAGATAAAGAGTTGTTTGCGAA-3’, annealing temperature 59° C and 34 cycles on a PTC-200 ThermalCycler (Biorad, Hercules, CA, USA)). Purification of the PCR product was performed with an Illustra™ GFX™ PCR Purification Kit (GE Healthcare, Little Chalfont, Buckinghamshire, UK). Sequence reactions were performed using BigDye v3.1 (Applied Biosystems, Forster City, CA, USA) and analyzed on an ABI PRISM 3100 Automated Sequencer (Applied Biosystems, Forster City, CA, USA). Sequence data were analyzed with the DNASTAR SeqMan® software (DNASTAR Inc., Madison, WI, USA).

### Statistical analyses

Demographic statistics were performed with SYSTAT11 (SYSTAT Software GmbH, Erkrath, Germany). Pearson χ^2^-statistics were used for sex, FH and smoking history; and Mann–Whitney U test for AAS.

Genetic association analyses were performed using HelixTree 6.3 (Golden Helix, Bozeman, MT, USA). All tag SNPs were analyzed for deviation from Hardy–Weinberg equilibrium. Based on the significant results in the PD-Goth material [23], haplotypes were analyzed for associations with PD (GAAAA, GAGGG and GAAAG in the haplotype window consisting of tag SNPs 2 – 6, AGC in the window consisting of promoter SNPs 1 – 3 and G**AGC**AAAA, G**AGA**AGGG and G**AGC**AAAG in the window consisting of all eight SNPs combined, see Figure 1 and Table 2 for SNP overview). A detailed description of how the haplotype windows were identified is given in our previous study [23], where the identified windows were corrected using permutations tests with 10 000 permutations and p_c_-values of ≤ 0.05 were considered statistically significant.

With meta-analysis sample sizes > 2000 individuals (after correction for unequal sample sizes) and a desired power of 80%, we can detect standardized differences down to approximately 0.13 in this study [33], which means that all the detected significances in risk and AAO reported in our previous paper [23] can be detected here at the applied significance level of 0.05.

SNP and haplotype associations were analyzed using logistic or linear haplotype regression, where each haplotype was analyzed individually relative to all the other haplotypes together. Sex was the only covariate available in all materials. In order to maximize sample size and power of the analyses and to make the results comparable between the independent materials, only sex was included as covariate in our model (except for risk of PD in the Polish material where the number of males and females were identical in the patient and control groups). However, when data was available and statistically relevant (see our previous study [23] for a detailed description of how statistically relevant covariates were selected), the effects of sex, FH and smoking habits were evaluated in the meta-analyses to assure that exclusion of covariates did not affect the outcomes. Haplotype frequencies were estimated in each material individually using the EM algorithm [34] allowing imputation to compensate for missing genotypes.

Meta-analyses were performed using inverse-variance weighting. In attempts to replicate our previously observed associations, the five replication materials were analyzed for associations of *NFE2L2* with PD risk and AAO. One-sided p-values ≤ 0.05 were considered statistically significant. In the exploratory meta-analyses for individual SNP associations with PD risk and AAO, all six materials were included in the analyses. Two-sided p-values ≤ 0.05 were considered statistically significant.

### Ethics

The study was approved by ethics committees in each respective country (Sweden Gothenburg: the regional ethics committee at University of Gothenburg, Sweden; Italy: the ethical committee at the University Hospital Mater Domini, Catanzaro, Italy; Sweden Linköping: the regional ethics committee at University of Linköping, Sweden; Malta: the research ethics committee at the University of Malta, Malta; Poland: the ethics committee of the Pomeranian Medical University, Szczecin, Poland; Germany: the ethics committee of the University of Lübeck). This study was in compliance with the Helsinki Declaration of 1975 and written informed consent was obtained from all participants.

## Results

### Demographics

*PD-Goth:* Patients and control subjects were similar in age, FH and current smoking status, but differed significantly in the distributions of sex and ever-smoker status. *Italy:* Patients and control subjects differed significantly in AAS and sex. No information was available regarding FH or smoking habits for the control subjects. *PD-Link:* Patients and control subjects did not differ with respect to FH, but differed significantly in AAS, sex and smoking status. *Malta:* Patients and control subjects were similar in AAS, sex and current-smoker status, but differed significantly with respect to FH and ever-smoker status. *Poland:* Patients and control subjects were matched in sex, but differed significantly in AAS due to the study design. No information was available regarding PD FH or smoking habits for either PD patients or control subjects. *Germany:* Patients and control subjects were similar regarding sex frequencies, but differed significantly in AAS. No information was available regarding PD FH or smoking habits for either PD patients or control subjects.

In general, all centers had a higher percentage of males relative to females amongst the patients (57.0 – 62.4%). Amongst the controls there was a wider range (36.8 – 62.9%) due to different study designs. Similarly, the average AAS was more similar between the patient groups (63.7 – 71.4 years) than between the control groups (57.0 – 72.9 years). The Polish material stood out, since the controls were older than the patients as a result of the study design. The average AAO varied between 55.2 and 64.1 years and the average time from AAO until AAS ranged from 6.4 to 9.2 years between the different patient groups. Where data was provided, FH of PD was always more common in the patient groups than in the controls, though this difference did not always reach statistical significance. As expected, smoking (ever smokers) were more common in all patient groups than in the control groups, whenever data was available. All demographics are given in Table 1. Frequencies for the studied *NFE2L2* SNPs and haplotypes in patients and controls are summarized in Additional file 1: Table S1 and Additional file 2: Table S2, respectively.

### Tag SNP genotyping

None of the studied SNPs had a Bonferroni-corrected Hardy-Weinberg equilibrium p-value of < 0.001. The overall call rate was > 95%.

### Haplotype associations

**Figure 2:**
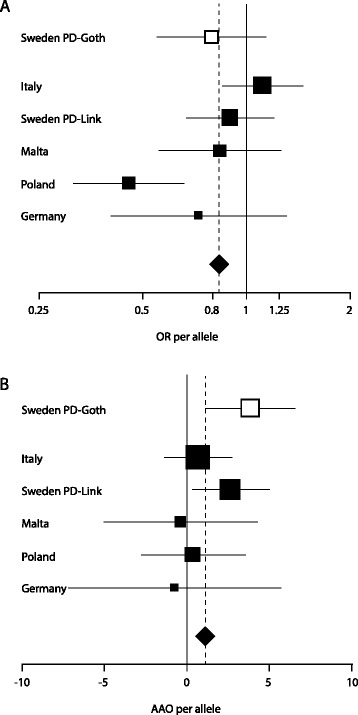
**Forest plot showing the** ***NFE2L2*** **haplotype GAGCAAAA associations with decreased risk (A) and later onset (B) of PD in meta-analysis including the five replication materials: Italy, Sweden PD-Link, Malta, Poland and Germany (filled squares).** The previous finding from the Swedish PD-Goth discovery study is also shown (empty squares), though the material was not included in the meta-analysis. The areas of the squares are proportional to the meta-analysis weights (inverse variance).

In accordance with the findings in the first study, meta-analyses of *NFE2L2* haplotypes including five independent replication patient-control materials showed a protective effect of the two haplotypes G**AGC**AAAA and GAAAA on PD. Haplotype G**AGC**AAAA, consisting of the five tag SNPs and the three functional promoter SNPs (SNPs 2, P1, P2, P3, 3, 4 ,5 and 6 according to gene direction, for rs numbers see Table 2), was associated with both decreased risk (OR = 0.8 per allele, p = 0.012) (Table 3, Figure 2A) and later PD onset (+1.1 years per allele, p = 0.048) (Table 4, Figure 2B). Haplotype GAAAA, consisting of tag SNPs 2 – 6 only, was associated with later PD onset (+1.3 years per allele, p = 0.024) (Table 4) and showed a tendency towards association with decreased PD risk (OR = 0.9 per allele, p = 0.052) (Table 3).

**Table 3:** **Replication of haplotype associations with risk of PD**

		**Sweden PD-Goth**	**Italy**	**Sweden PD-Link**	**Malta**	**Poland**	**Germany**	**Meta-analysis** ^**2**^	
**SNP** ^**1**^	**Haplotypes**	**OR/allele (CI)**	**OR/allele (CI)**	**OR/allele (CI)**	**OR/allele (CI)**	**OR/allele (CI)**	**OR/allele (CI)**	**OR/allele (CI)**	**p-value**
2, 3, 4, 5, 6	GAAAA	0.8 (0.5 – 1.1)	1.1 (0.9 – 1.5)	0.9 (0.7 – 1.2)	0.8 (0.6 – 1.3)	0.7 (0.5 – 0.9)	0.7 (0.4 – 1.3)	0.88 (0.76 – 1.03)	0.052
	GAGGG	2.1 (1.2 – 3.8)	1.2 (0.8 – 1.7)	0.9 (0.5 – 1.3)	0.7 (0.4 – 1.3)	1.0 (0.6 – 1.6)	1.1 (0.5 – 2.5)	1.00 (0.81 – 1.24)	0.50
	GAAAG	2.8 (1.0 – 7.7)	2.8 (1.1 – 6.8)	0.4 (0.1 – 1.2)	0.0 (0.0 – 2.9 × 10^18^)	0.5 (0.2 – 1.7)	0.0 (0.0 – 21.6)	0.98 (0.54 – 1.77)	0.53
P1, P2, P3	AGC	1.0 (0.7 – 1.3)	1.1 (0.9 – 1.3)	0.8 (0.6 – 1.1)	1.0 (0.7 – 1.3)	0.8 (0.6 – 1.0)	0.9 (0.5 – 1.5)	0.92 (0.81 – 1.04)	0.09
2, P1, P2, P3, 3, 4, 5, 6	G**AGC**AAAA	0.8 (0.6 – 1.1)	1.1 (0.9 – 1.5)	0.9 (0.7 – 1.2)	0.8 (0.6 – 1.3)	0.5 (0.3 – 0.7)	0.7 (0.4 – 1.3)	0.84 (0.72 – 0.98)	**0.012**
	G**AGA**AGGG	2.4 (1.3 – 4.5)	1.2 (0.9 – 1.8)	0.9 (0.6 – 1.4)	0.8 (0.4 – 1.4)	0.6 (0.3 – 1.0)	1.1 (0.5 – 2.5)	0.95 (0.76 – 1.18)	0.68
	G**AGC**AAAG	2.9 (1.0 – 8.4)	2.8 (1.1 – 6.8)	0.4 (0.1 – 1.2)	0.0 (0.0 – 3.3 × 10^36^)	0.4 (0.1 – 1.5)	0.0 (0.0 – 29.2)	0.95 (0.52 – 1.72)	0.57

Odds ratios (OR) are presented as absolute numbers (95% CI). Sex was used as covariate for all materials except for Poland, where the number of males and females are identical in patients and controls. Statistically significant p-values are highlighted in bold.

^1^For SNP locations see Figure 1 and Table 2.

^2^The meta-analysis was calculated using inverse-variance weighting of effects with one-sided p-values and included all materials except Sweden PD-Goth, since it is the discovery material on which the haplotype selection was based.

**Table 4:** **Replication of haplotype associations with AAO of PD**

		**Sweden PD-Goth**	**Italy**	**Sweden PD-Link**	**Malta**	**Poland**	**Germany**	**Meta-analysis** ^**2**^	
**SNP** ^**1**^	**Haplotypes**	**Years/allele (CI)**	**Years/allele (CI)**	**Years/allele (CI)**	**Years/allele (CI)**	**Years/allele (CI)**	**Years/allele (CI)**	**Years/allele (CI)**	**p-value**
2, 3, 4, 5, 6	GAAAA	+4.1 (+1.3 – +6.8)	+0.6 (−1.5 – +2.6)	+2.8 (+0.5 – +5.1)	−0.4 (−5.0 – +4.3)	+1.2 (−1.6 – +4.0)	−0.2 (−6.8 – +6.5)	+1.28 (−0.01 – +2.54)	**0.024**
	GAGGG	−0.9 (−4.6 – +32.8)	−0.4 (−2.9 – +2.2)	+2.1 (−1.6 – +6.0)	+0.5 (−7.0 – +8.1)	−0.7 (−4.2 – +2.8)	+1.3 (−5.4 – +8.0)	+0.19 (−1.50 – +1.89)	0.59
	GAAAG	−3.6 (−9.1 – +2.0)	−0.2 (−5.7 – +5.4)	+1.0 (−8.7 – +10.7)	+253.1 (−483.1 – +989.3)	−1.2 (−10.8 – +8.4)	−60.9 (−221.2 – +99.3)	−0.16 (−4.46 – +4.14)	0.47
P1, P2, P3	AGC	+1.4 (−0.7 – +3.6)	+0.8 (−0.8 – +2.4)	−0.1 (−2.1 – +1.9)	+0.3 (−3.1 – +3.7)	−0.0 (−2.2 – +2.1)	−1.6 (−6.2 – +2.9)	+0.22 (−0.79 – +1.23)	0.33
2, P1, P2, P3, 3, 4, 5, 6	G**AGC**AAAA	+3.8 (+1.1 – +6.5)	+0.7 (−1.4 – +2.7)	+2.6 (+0.3 – +5.0)	−0.4 (−5.0 – +4.3)	+0.4 (−2.8 – +3.6)	−0.7 (−7.2 – +5.7)	+1.10 (−0.20 – +2.40)	**0.048**
	G**AGA**AGGG	−0.8 (−4.6 – +2.9)	−0.3 (−2.9 – +2.2)	+2.1 (−1.7 – +6.0)	−0.5 (−7.0 – +8.0)	+0.5 (−3.7 – +4.8)	+1.3 (−5.4 – +8.0)	+0.51 (−1.26 – +2.28)	0.71
	G**AGC**AAAG	−3.0 (−8.6 – +2.6)	+0.2 (−5.4 – +5.8)	+0.8 (−9.1 – +10.6)	−2817.1 (−5873.3 – +239.1)	−0.6 (−11.4 – +10.2)	−134.6 (−321.2 – +51.9)	+0.09 (−4.35 – +4.52)	0.52

Effects on AAO are given as absolute numbers (95% CI). Sex was used as covariate for all materials. Statistically significant p-values are highlighted in bold.

^1^For SNP locations see Figure 1 and Table 2.

^2^The meta-analysis was calculated using inverse-variance weighting of effects with one-sided p-values and included all materials except Sweden PD-Goth, since it is the discovery material on which the haplotype selection was based.

None of the haplotypes G**AGA**AGGG, GAGGG, G**AGC**AAAG or GAAAG in the same two windows, all associated with increased risk in the PD-Goth material in the first study, was significantly associated with PD in the meta-analyses. The promoter haplotype AGC alone was not associated with PD in meta-analysis (Tables 3 and 4).

### Individual SNP associations

Our previous protective association of the A allele of tag SNP 6 (rs10183914) in the PD-Goth discovery study [23] could not be replicated in the meta-analysis including the five replication materials (+0.3 year per A allele, p = 0.23).

Exploratory meta-analyses of *NFE2L2* individual SNPs, including all six materials, showed no significant associations with risk of PD (Table 5).

**Table 5:** **Individual SNP associations with risk of PD**

		**Sweden PD-Goth**	**Italy**	**Sweden PD-Link**	**Malta**	**Poland**	**Germany**	**Meta-analysis** ^**2**^	
**SNP** ^**1**^	**Genotype**	**OR/allele (CI)**	**OR/allele (CI)**	**OR/allele (CI)**	**OR/allele (CI)**	**OR/allele (CI)**	**OR/allele (CI)**	**OR/allele (CI)**	**p-value**
2 – rs7557529	G > A	0.9 (0.7 – 1.2)	0.8 (0.7 – 1.0)	1.2 (1.0 – 1.6)	1.5 (1.1 – 2.0)	1.3 (1.0 – 1.8)	1.0 (0.6 – 1.7)	1.08 (0.96 – 1.21)	0.18
P1 – rs35652124	A > G	0.8 (0.6 – 1.2)	0.7 (0.6 – 0.9)	1.3 (1.0 – 1.7)	0.9 (0.6 – 1.3)	1.3 (1.0 – 1.8)	1.1 (0.6 – 1.8)	0.99 (0.87 – 1.12)	0.86
P2 – rs6706649	G > A	0.9 (0.6 – 1.4)	1.3 (0.9 – 1.8)	1.1 (0.7 – 1.5)	1.6 (1.0 – 2.4)	1.0 (0.7 – 1.5)	0.9 (0.4 – 2.0)	1.14 (0.96 – 1.35)	0.14
P3 – rs6721961	C > A	1.8 (1.1 – 3.0)	1.1 (0.8 – 1.5)	0.9 (0.6 – 1.4)	0.7 (0.4 – 1.1)	1.0 (0.7 – 1.6)	1.3 (0.7 – 2.6)	1.07 (0.90 – 1.27)	0.45
3 – rs2886161	A > G	0.8 (0.6 – 1.2)	0.7 (0.5 – 0.9)	1.3 (1.0 – 1.7)	1.0 (0.7 – 1.4)	1.3 (1.0 – 1.8)	1.1 (0.6 – 1.8)	0.98 (0.87 – 1.12)	0.81
4 – rs1806649	G > A	1.0 (0.7 – 1.3)	1.2 (0.9 – 1.5)	0.8 (0.6 – 1.1)	0.8 (0.6 – 1.2)	0.7 (0.5 – 0.9)	0.7 (0.4 – 1.2)	0.88 (0.77 – 1.01)	0.07
5 – rs2001350	A > G	2.0 (1.1 – 3.6)	1.3 (1.0 – 1.8)	0.9 (0.6 – 1.3)	0.8 (0.4 – 1.4)	0.9 (0.6 – 1.4)	1.0 (0.5 – 2.1)	1.10 (0.91 – 1.34)	0.31
6 – 10183914	G > A	0.8 (0.6 – 1.2)	1.1 (0.9 – 1.4)	0.8 (0.6 – 1.1)	1.0 (0.7 – 1.4)	0.8 (0.6 – 1.1)	0.9 (0.6 – 1.6)	0.93 (0.82 – 1.05)	0.22

Odds ratios (OR) are given for the minor allele and presented as absolute numbers (95% CI). Sex was used as covariate for all materials except for Poland, where the number of males and females are identical in patients and controls.

^1^For SNP locations see Figure 1 and Table 2.

^2^The meta-analysis was calculated using inverse-variance weighting of effects with two-sided p-values, including all six materials.

Exploratory meta-analyses of *NFE2L2* individual SNPs with AAO, including all six materials, showed associations with four SNPs; tag SNP 2 (rs7557529, −1.0 year per A allele, p = 0.042), promoter SNP 1 (rs35652124, −1.1 year per G allele, p = 0.045), tag SNP 3 (rs2886161, −1.2 year per G allele, p = 0.021) and tag SNP 4 (rs1806649, +1.2 year per A allele, p = 0.029) (Table 6).

**Table 6:** **Individual SNP associations with AAO of PD**

		**Sweden PD-Goth**	**Italy**	**Sweden PD-Link**	**Malta**	**Poland**	**Germany**	**Meta-analysis** ^**2**^	
**SNP** ^**1**^	**Genotype**	**Years/allele (CI)**	**Years/allele (CI)**	**Years/allele (CI)**	**Years/allele (CI)**	**Years/allele (CI)**	**Years/allele (CI)**	**Years/allele (CI)**	**p-value**
2 – rs7557529	G > A	−1.7 (−3.9 – +0.6)	−0.4 (−2.0 – +1.3)	−2.2 (−4.3 – -0.1)	−0.8 (−4.7 – +3.1)	−0.2 (−2.4 – +2.0)	−0.8 (−6.0 – +4.3)	−0.99 (−1.94 – -0.03)	**0.042**
P1 – rs35652124	A > G	−2.1 (−4.5 – +0.3)	−1.3 (−3.3 – +0.7)	−1.8 (−4.1 – +0.5)	−1.6 (−5.8 – +2.6)	+0.2 (−2.0 – +2.4)	+2.8 (−2.3 – +7.8)	−1.07 (−2.12 – -0.02)	**0.045**
P2 – rs6706649	G > A	+1.4 (−1.8 – +4.7)	+1.4 (−0.9 – +3.8)	+1.4 (−1.5 – +4.2)	−0.0 (−5.2 – +5.2)	−0.6 (−3.7 – +2.5)	−1.7 (−9.5 – +6.0)	+0.85 (−0.49 – +2.19)	0.22
P3 – rs6721961	C > A	−1.5 (−5.0 – +2.0)	−0.7 (−2.7 – +1.3)	+2.1 (−1.4 – +5.6)	+2.7 (−3.2 – +8.6)	+0.3 (−2.8 – +3.3)	+0.0 (−6.0 – +6.1)	−0.81 (−2.03 – +0.41)	0.19
3 – rs2886161	A > G	−2.4 (−4.8 – -0.1)	−1.4 (−3.4 – +0.6)	−2.0 (−4.3 – +0.3)	−1.5 (−5.6 – +2.7)	+0.0 (−2.2 – +2.2)	+2.7 (−2. 4 – +7.8)	−1.23 (−2.27 – -0.18)	**0.021**
4 – rs1806649	G > A	+2.7 (+0.2 – +5.1)	+0.4 (−1.5 – +2.4)	+2.7 (+0.4 – +4.9)	−2.4 (−7.0 – +2.1)	+1.0 (−1.7 – +3.7)	−3.6 (−9.5 – +2.3)	+1.20 (+0.12 – +2.28)	**0.029**
5 – rs2001350	A > G	−0.6 (−4.3 – +3.0)	−0.8 (−3.1 – +1.6)	+1.8 (−1.9 – +5.6)	+1.8 (−5.1 – +8.8)	−0.7 (−4.1 – +2.8)	+1.1 (−5.7 – +7.8)	−0.11 (−1.59 – +1.37)	0.88
6 – 10183914	G > A	+3.7 (+1.3 – +6.2)	+0.3 (−1.3 – +2.0)	+1.5 (−0.6 – +3.7)	−0.4 (−4.2 – +3.3)	−0.1 (−2.4 – +2.3)	−1.4 (−6.2 – +3.5)	+0.95 (−0.04 – +1.94)	0.06

Effects on AAO are given for the minor allele and presented as absolute numbers (95% CI). Sex was used as covariate for all materials. Statistically significant p-values are highlighted in bold.

^1^For SNP locations see Figure 1 and Table 2.

^2^The meta-analysis was calculated using inverse-variance weighting of effects with two-sided p-values, including all six materials.

## Discussion

Since oxidative stress is implicated in the pathogenesis of PD [2,3] and Nrf2 is a central protein in the cellular defense against oxidative stress, genetic variation affecting the efficiency of Nrf2 could contribute to the disease. Here, based on the previous results from our group [23], we performed meta-analyses on five independent patient-control materials to investigate the effects of seven haplotypes in the Nrf2-encoding gene *NFE2L2* on risk and AAO of PD. In addition, the included SNPs were analyzed individually in exploratory meta-analyses including the PD-Goth discovery material. To our knowledge, with 1038 PD patients and 1600 control subjects included, this is the most extensive attempt yet to investigate the impact of genetic variation in *NFE2L2* on PD risk and AAO.

Meta-analyses of three *NFE2L2* haplotypes containing five consecutive tag SNPs and three functional promoter SNPs showed association of haplotype G**AGC**AAAA with both decreased risk and later onset of PD (Tables 3 and 4, Figure 2). This supports our previously observed protective effect of this haplotype, which was associated with later onset of PD in the PD-Goth discovery material and decreased risk of PD in the Polish replication material in the first study [23]. In addition, meta-analyses of the three previously associated *NFE2L2* haplotypes in the window including the five tag SNPs only (tag SNPs 2 – 6) showed association of haplotype GAAAA with later onset of PD. This haplotype also had a tendency towards association with decreased risk of PD. Obviously, G**AGC**AAAA and GAAAA are highly influenced by one another and should not be considered separate haplotypes. The promoter haplotype AGC is in LD with the PD-associated haplotype GAAAA (r^2^ = 0.4) [23] and the PD-associated combined haplotype G**AGC**AAAA contains the fully functional promoter haplotype AGC [24,25]. This suggests that the associations could be driven by genetic variation in the *NFE2L2* promoter. However, the fact that the promoter haplotype AGC alone showed no association with PD in meta-analysis indicates that other parts of the gene are involved as well. This is also in accordance with a previous study on a Taiwanese material, where none of the *NFE2L2* promoter haplotypes were associated with PD [35].

Haplotypes G**AGA**AGGG, GAGGG, G**AGC**AAAG and GAAAG in the same two haplotype windows were all associated with increased risk of PD in the PD-Goth discovery material, but none of the associations could be replicated here. Likewise, the association of tag SNP 6 (rs10183914) with later PD onset in the PD-Goth discovery study could not be replicated in meta-analysis.

In line with our previous results, individual exploratory meta-analyses of the genotyped *NFE2L2* SNPs on patient and control subjects from six independent European centers showed no associations with risk of PD. However, in this study four *NFE2L2* SNPs were associated with AAO of PD in the exploratory meta-analyses. The minor alleles of tag SNP 2 (rs7557529), promoter SNP P1 (rs35652124) and tag SNP 3 (rs2886161) were all associated with one year earlier PD onset per allele. Tag SNP 3 of our study was previously included in a multiple candidate gene study, but was not significantly associated with PD [36]. Tag SNP 2 is in LD (r^2^ = 0.9) with SNP rs6726395 [23], which was previously found to increase the risk of PD in a first-tier, sib-pair whole-genome study of PD, though the association was not replicated in the second-tier case–control study [37]. The minor G allele of promoter SNP P1 has been shown to significantly decrease Nrf2 protein levels [24,25]. Tag SNP 2 and especially tag SNP 3 of our study are both in LD with the minor G allele of P1 (r^2^ = 0.4 and r^2^ = 0.9, respectively) (Figure 1), which could possibly explain the observed associations of these SNPs with earlier PD onset in our study. The minor A allele of tag SNP 4 (rs1806649) was instead protective and delayed onset of PD with one year per allele in meta-analysis. Consistent with the discussion above, this SNP was not in LD with SNP P1 (r^2^ = 0.1). Activation of Nrf2 with natural or synthetic substances have been shown to reduce oxidative stress and neurodegeneration in different PD models [10,11], which suggests that Nrf2 could be a suitable target for pharmacological intervention of PD. In this context, the associations of *NFE2L2* variants with AAO of PD are interesting.

Though haplotype G**AGC**AAAA showed a trend towards increased risk of PD in the Italian material when the materials were analyzed individually, this haplotype decreased the risk of PD in meta-analysis (Table 3, Figure 2A). Idiopathic PD is a multifactorial disease caused by several risk genes, possibly in combination with environmental factors. This may explain why the effect of a risk gene could be more obvious in some materials than in others. Also, study design as well as different genetic background in both PD patients and controls could affect the outcome of a multicenter study. Considering this, despite the relatively small effects in the meta-analyses, the replicated associations between genetic variation in the *NFE2L2* gene and idiopathic PD are interesting and should be investigated further in functional studies.

## Conclusions

Meta-analyses including five independent patient-control materials from different European centers confirmed protective effects of *NFE2L2* haplotypes G**AGC**AAAA and GAAAA against PD. In addition, exploratory meta-analyses revealed four individual SNPs affecting the AAO of PD, including a functional SNP located in the *NFE2L2* promoter. In summary, our data support the hypothesis that variation in the Nrf2-encoding gene *NFE2L2* may contribute to the pathogenesis of idiopathic PD.

## Additional files

Additional file 1: Table S1. Individual SNP frequencies in PD patients and control subjects. Additional file 2: Table S2. Haplotype frequencies in PD patients and control subjects.

## Abbreviations

AAD: Age at diagnosis
AAFS: Age at first symptom
AAO: Age at onset
AAS: Age at sampling
ARE: Antioxidant response element
DA: Dopamine
DAergic: Dopaminergic
FH: Family history
HO-1: Heme oxygenase 1
iPSCs: Induced pluripotent stem cells
Keap1: Kelch-like ECH-associated protein 1
LD: Linkage disequilibrium
MPTP: 1-methyl-4-phenyl-1,2,3,6-tetrahydropyridine
*NFE2L2*: NF-E2-like 2
Nrf2: Nuclear factor erythroid 2 (NF-E2) related factor 2
NQO1: NAD(P)H dehydrogenase, quinone 1
PD: Parkinson’s disease
SNP: Single nucleotide polymorphism
SN: Substantia nigra
