# Supplementary material for: Genetic associations of Nrf2-encoding NFE2L2 variants with Parkinson’s disease – a multicenter study
Source: BMC Med Genet. 2014 Dec 12;15:131. doi: 10.1186/s12881-014-0131-4 (PMC4335439; doi:10.1186/s12881-014-0131-4)
Supplement: Additional file 1: Table S1. — Individual SNP frequencies in PD patients and control subjects. [file 12881_2014_131_MOESM1_ESM.pdf]

Additional file 1: Table S1 Individual SNP frequencies in PD patients and control subjects

		Sweden PD-Goth		Italy		Sweden PD-Link		Malta		Poland		Germany	
		PD	Control	PD	Control	PD	Control	PD	Control	PD	Control	PD	Control
**SNP**	**Genotype**												
	GG	40 (24.4)	42 (22.1)	77 (24.3)	102 (23.9)	29 (15.1)	91 (24.7)	16 (16.5)	82 (26.5)	37 (19.8)	55 (30.7)	10 (20.4)	22 (29.7)
**2 – rs7557529**	AG	85 (51.8)	92 (48.4)	173 (54.6)	208 (48.7)	105 (54.7)	175 (47.4)	48 (49.5)	151 (48.9)	90 (48.1)	75 (41.9)	26 (53.1)	27 (36.5)
	AA	39 (23.8)	56 (29.5)	67 (21.1)	117 (27.4)	58 (30.2)	103 (27.9)	33 (34.0)	76 (24.6)	60 (32.1)	49 (27.4)	13 (26.5)	25 (33.8)
	GG	15 (9.3)	13 (7.1)	9 (2.8)	35 (8.0)	15 (7.7)	35 (9.4)	6 (6.1)	25 (8.1)	26 (13.6)	17 (9.1)	6 (10.7)	12 (16.2)
**P1 – rs35652124**	AG	58 (35.8)	89 (48.3)	132 (41.5)	185 (42.2)	110 (56.7)	162 (43.3)	39 (39.8)	122 (39.4)	77 (40.3)	67 (35.8)	30 (53.6)	30 (40.5)
	AA	89 (54.9)	82 (44.6)	177 (55.7)	218 (49.8)	69 (35.6)	177 (47.3)	53 (54.1)	163 (52.5)	88 (46.1)	103 (55.1)	20 (35.7)	32 (43.3)
	AA	4 (2.5)	3 (1.6)	3 (0.9)	4 (0.9)	4 (2.1)	8 (2.1)	5 (5.1)	6 (1.9)	6 (3.1)	2 (1.1)	1 (1.8)	3 (4.1)
**P2 – rs6706649**	AG	33 (20.4)	45 (24.5)	81 (25.5)	89 (20.3)	44 (22.7)	76 (20.3)	26 (26.5)	62 (20.0)	35 (18.3)	42 (22.5)	7 (12.5)	8 (10.8)
	GG	125 (77.1)	136 (73.9)	234 (73.6)	345 (78.8)	146 (75.2)	291 (77.6)	67 (68.4)	242 (78.1)	150 (78.6)	143 (76.4)	48 (85.7)	63 (85.1)
	AA	1 (0.6)	1 (0.5)	13 (4.1)	8 (1.8)	0 (0.0)	6 (1.6)	1 (1.0)	8 (2.6)	6 (3.1)	2 (1.1)	2 (3.6)	2 (2.7)
**P3 – rs6721961**	AC	39 (24.1)	27 (14.7)	78 (24.5)	112 (25.6)	37 (19.1)	65 (17.3)	19 (19.4)	77 (24.8)	38 (19.9)	43 (23.0)	14 (25.0)	15 (20.3)
	CC	122 (75.3)	156 (84.8)	227 (71.4)	318 (72.6)	157 (80.9)	304 (81.1)	78 (79.6)	225 (72.6)	147 (77.0)	142 (75.9)	40 (71.4)	57 (77.0)
	GG	16 (9.7)	15 (7.9)	9 (2.8)	34 (7.9)	15 (7.8)	35 (9.5)	6 (6.1)	25 (8.1)	27 (14.4)	17 (9.4)	6 (11.3)	12 (16.2)
**3 – rs2886161**	AG	59 (35.8)	91 (47.9)	133 (41.6)	187 (43.2)	108 (56.3)	159 (43.1)	41 (41.4)	120 (38.7)	75 (40.1)	67 (37.0)	27 (50.9)	29 (39.2)
	AA	90 (54.5)	84 (44.2)	178 (55.6)	212 (48.9)	69 (35.9)	175 (47.4)	52 (52.5)	165 (53.2)	85 (45.5)	97 (53.6)	20 (37.8)	33 (44.6)
	AA	14 (8.5)	15 (7.9)	15 (4.8)	20 (4.7)	13 (6.8)	28 (7.6)	5 (5.1)	15 (4.9)	9 (4.8)	23 (12.7)	2 (3.8)	8 (11.0)
**4 – rs1806649**	AG	65 (39.4)	80 (42.1)	113 (35.9)	137 (32.5)	61 (31.8)	142 (38.4)	29 (29.6)	113 (36.6)	60 (32.3)	61 (33.7)	17 (32.1)	24 (32.9)
	GG	86 (52.1)	95 (50.0)	187 (59.3)	265 (62.8)	118 (61.4)	200 (54.0)	64 (65.3)	181 (58.5)	117 (62.9)	97 (53.6)	34 (64.1)	41 (56.1)
	GG	1 (0.6)	0 (0.0)	4 (1.3)	5 (1.2)	0 (0.0)	5 (1.4)	0 (0.0)	4 (1.3)	4 (2.1)	0 (0.0)	2 (3.7)	0 (0.0)
**5 – rs2001350**	AG	35 (21.2)	23 (12.1)	80 (25.1)	85 (19.8)	32 (16.7)	60 (16.2)	17 (16.8)	57 (18.4)	28 (14.7)	39 (20.9)	8 (14.8)	17 (23.0)
	AA	129 (78.2)	167 (87.9)	235 (73.6)	339 (79.0)	160 (83.3)	305 (82.4)	84 (83.2)	249 (80.3)	158 (83.2)	148 (79.1)	44 (81.5)	57 (77.0))
	AA	14 (8.5)	25 (13.2)	32 (10.1)	39 (9.3)	17 (9.0)	47 (12.7)	14 (14.9)	37 (12.7)	20 (10.8)	28 (15.0)	7 (13.0)	9 (12.2)
**6 – 10183914**	AG	81 (49.1)	89 (46.8)	139 (44.0)	184 (43.8)	83 (43.9)	166 (44.7)	37 (39.4)	130 (44.7)	73 (39.2)	73 (39.0)	20 (37.0)	30 (40.5)
	GG	70 (42.4)	76 (40.0)	145 (45.9)	197 (46.9)	89 (47.1)	158 (42.6)	43 (45.7)	124 (42.6)	93 (50.0)	86 (46.0)	27 (50.0)	35 (47.3)

SNP frequencies given as absolute numbers (%).
