# Supplementary material for: Genetic associations of Nrf2-encoding NFE2L2 variants with Parkinson’s disease – a multicenter study
Source: BMC Med Genet. 2014 Dec 12;15:131. doi: 10.1186/s12881-014-0131-4 (PMC4335439; doi:10.1186/s12881-014-0131-4)
Supplement: Additional file 2: Table S2. — Haplotype frequencies in PD patients and control subjects. [file 12881_2014_131_MOESM2_ESM.pdf]

Additional file 2 Table S2 Haplotype frequencies in PD patients and control subjects

		Sweden PD-Goth		Italy		Sweden PD-Link		Malta		Poland		Germany	
		**PD**	**Control**	**PD**	**Control**	**PD**	**Control**	**PD**	**Control**	**PD**	**Control**	**PD**	**Control**
**SNPs**	**Haplotypes**												
**2, 3, 4, 5, 6**	GAAAA	21.3	26.7	19.1	19.0	21.0	23.3	20.0	22.7	18.9	26.6	19.6	24.0
	GAGGG	10.9	5.5	11.2	9.6	7.8	9.0	8.4	10.1	9.3	10.5	11.3	10.0
	GAAAG	5.4	1.7	2.5	1.2	1.1	3.0	<1.0	<1.0	1.4	2.5	<1.0	2.9
**P1, P2, P3**	AGC	47.5	47.0	46.4	45.4	41.0	46.4	44.9	45.3	40.8	48.1	38.4	41.2
**2, P1, P2, P3, 3, 4, 5, 6**	G**AGC**AAAA	22.1	26.3	19.3	18.8	21.1	23.3	20.0	22.6	12.8	26.9	19.7	24.7
	G**AGA**AGGG	10.6	5.0	10.8	9.0	7.7	8.5	8.4	9.9	5.7	10.1	10.7	9.8
	G**AGC**AAAG	4.8	1.5	2.6	1.1	1.1	3.0	<1.0	<1.0	1.0	2.6	<1.0	2.8

Haplotype frequencies (%) estimated using the EM-algorithm [34].
